# Pro-Atherogenic and Pro-Oxidant Diets Influence Semen and Blood Traits of Rabbit Bucks

**DOI:** 10.3390/antiox12101880

**Published:** 2023-10-19

**Authors:** Simona Mattioli, Elisa Angelucci, Alessandro Dal Bosco, Cinzia Signorini, Lakamy Sylla, Luigia Bosa, Giulia Collodel, Thierry Durand, Jean-Marie Galano, Camille Oger, Cesare Castellini

**Affiliations:** 1 Department of Agricultural, Environmental and Food Science, University of Perugia, Borgo XX Giugno 74, 06124 Perugia, Italy; elisa.angelucci@unipg.it (E.A.); alessandro.dalbosco@unipg.it (A.D.B.); luigia.bosa@studenti.unipg.it (L.B.); cesare.castellini@unipg.it (C.C.); 2 Department of Molecular and Developmental Medicine, University of Siena, Policlinico Santa Maria alle Scotte, Viale Bracci, 14, 53100 Siena, Italy; cinzia.signorini@unisi.it (C.S.); giulia.collodel@unisi.it (G.C.); 3 Department of Veterinary Medicine, University of Perugia, Via S. Costanzo 4, 06126 Perugia, Italy; lakamy.sylla@unipg.it; 4 Institut des Biomolécules Max Mousseron (IBMM), UMR, CNRS, Université de Montpellier, ENSCM, 5247 Montpellier, France; thierry.durand@umontpellier.fr (T.D.); jean-marie.galano@umontpellier.fr (J.-M.G.); camille.oger@umontpellier.fr (C.O.)

**Keywords:** saturated fatty acids, polyunsaturated fatty acids, semen, rabbit, oxidation, inflammation

## Abstract

Many dietary factors can affect sperm traits. We compared the effect of diets rich in pro-oxidant (flaxseed oil) and pro-atherogenic (coconut oil) substances without added antioxidants on semen traits, using the rabbit as an animal model. Thirty rabbit bucks (8 months old) were fed three diets for 150 days: CNT (control) a standard diet; HA (high-atherogenic) standard diet + 3% coconut oil, and HO (high-oxidizing) standard diet + 3% flaxseed oil. Semen samples were collected weekly for the evaluation of qualitative traits (kinetics, viability) and the oxidative damage (MDA and cytokines). Blood was collected at the start (T0) and end (T8) of the experimental period for the assessment of the oxidative damage (MDA and isoprostanoids), lipid profile, and testosterone. A worsening of sperm kinetics and viability was recorded in the HA group. Lipid oxidation in seminal plasma, as well as isoprostanoids in blood (F_3_-IsoPs and F_4_-NeuroPs), increased in both the HO and HA groups. A high level of TNF-α, a marker of inflammatory status, was recorded in the seminal plasma of the HA group. The resulting outcomes were mainly attributable to the different fatty acid profiles (SFA vs. PUFA) of the diets, which modulated an inflammatory/oxidative response.

## 1. Introduction

Diet is one of the main factors that affects male fertility worldwide [1,2], together with environment and lifestyle (i.e., smoking cigarettes and cannabis, anabolic steroid use, excessive alcohol consumption, excessive exposure to high temperatures, age, environmental pollution, sedentary lifestyle, exposure to pesticides and toxins, radiofrequency electromagnetic radiation, cytotoxic drugs, cadmium) [2].

The effect of diet on sperm quality depends on both quantitative and qualitative aspects, such as the energy content of macronutrients (carbohydrates, proteins, and fats), and the fatty acid, carbohydrate, and protein profiles [1,2]. It is widely known that unhealthy hypercaloric diets and excessive intake of saturated (SFA) and *trans* fatty acids have a negative impact on sperm quality and may be directly associated with increased oxidative stress, which is the underlying cause of reproductive problems [3,4,5]. Indeed, an adequate intake of antioxidant molecules has been quite effective in the prevention and/or treatment of male infertility [6,7]. It has been shown that many dietary substances such as polyphenols (mainly flavonoids), carotenes (mainly lycopene, astaxanthin, and β-carotene), and vitamins (mainly vitamin E and C) isolated from fruits, vegetables, and edible plants modulate mitochondrial metabolism and biogenesis, as well as reactive oxygen species (ROS) homeostasis [8]. Such antioxidant molecules have been demonstrated to have positive effects on testicular mitochondrial function (ROS reduction) as it is a modulator of lipid peroxidation, antioxidant enzyme activities, and activity of the Krebs cycle [9].

Healthy dietary patterns are associated with better sperm quality, suggesting that nutritional interventions could have a key role in the preservation of male fertility [10,11]. 

In recent decades, in Western countries, a specific dietary pattern has been consolidated [12,13]. The Western diet is characterized by a high intake of animal proteins, SFAs, and simple sugars, as well as a low supply of dietary fiber and polyunsaturated fatty acids (PUFAs). Furthermore, within PUFAs, the high intake of fatty acids (FAs) from n-6 series (i.e., C18:2, linoleic acid-LA; C20:4, arachidonic acid-ARA) is due to the wide diffusion of these FAs in common foods, compared to n-3 series (C18:3, α-linolenic acid—ALA; C20:5, eicosapentaenoic acid—EPA; C22:5, docosapentaenoic acid—DPA and C22:6, docosahexaenoic acid—DHA). Therefore, a recent report [14] from the Food and Agriculture Organization (FAO) established that the n-6/n-3 ratio in human foods should be close to 4:1, in contrast to the unbalanced ratio of 20–10:1 in Western diets [13].

Additionally, the n-3 and n-6 PUFA derivatives are metabolically and functionally distinct, and have the opposite physiological functions: n-6 PUFA metabolites (two-series prostaglandin-PG and thromboxane-TX and four-series leukotrienes-LT) have proinflammatory, prothrombotic, and aggregatory properties, which increase blood viscosity, vasospasm, and vasoconstriction [15]. On the contrary, n-3 metabolites (three-series PG and TX and five-series LT) have anti-inflammatory, anti-proliferative, and anti-atherosclerotic activity [16]. A balance between these molecules is suggested because the free radical-mediated peroxidation of both PUFA series originates prostaglandin-like products, namely isoprostanoids are connected with lipid oxidation [17,18]. The oxidation of arachidonic acid (LC-PUFA n-6) produces F_2_-isoprostanes (F_2_-IsoPs), whereas the oxidation of EPA and DHA (n-3 LC-PUFA) generates F_3_-isoprostanes (F_3_-IsoPs) and F_4_-neuroprostanes (F_4_-NeuroPs), respectively [18].

As mentioned above, the Western diet may be linked to an increase in the deterioration of semen quality [19]. A study of an Iranian group identified a decline in sperm count in the last 50 years, which, if it continues, could lead to a drastic reduction of sperm count by 2050, with widespread fertility problems [20,21]. 

However, human studies are generally undertaken on heterogeneous groups of fertile men because it is difficult to select participants with established dietary habits and environmental and health conditions; thus, to design robust experimental trials, animal models are often used. The rabbit is very useful in longitudinal studies on male fertility because semen collection does not require the killing of the animals and the sperm traits are easily standardized [22,23] and comparable to humans. 

Our previous research assessed the positive effect of dietary n-3 PUFA on rabbit semen quality [17] and the importance of antioxidants (tocopherols) to balance the oxidative thrust induced by PUFAs [24]. In the present research, we compared blood and semen traits of rabbits fed diets rich in pro-oxidant (flaxseed oil) and pro-atherogenic (coconut oil) substances without balancing the antioxidants of the diets, underlining the connection between the nature of dietary fatty acids and the development of inflammatory conditions.

## 2. Materials and Methods

### 2.1. Animals and Experimental Design

Thirty rabbits (8 months old) were selected and divided into three experimental groups (10 per group) and fed different diets (Table 1) for 110 days (about 50 days of adaptation and 60 days for the experimental period). The control group (CNT) was fed *ad libitum* the standard diet (Table 1), the HA group (high-atherogenic) was fed a standard diet supplemented with 3% coconut oil (CocoNativo Bio, Monte Nativo, Italy), and the HO group (high-oxidative) was fed a standard diet supplemented with 3% flaxseed oil (flaxseed oil 100%, Bleu&Marine Bretania Cosmetics, Alhaurín de la Torre, France). Digestible energy was estimated by Maertens et al. [25]. The rabbits were housed according to Boiti et al. [22]. 

This study was conducted in accordance with the Guiding Principles in the use of animals and approved by the Animal Ethics Monitoring Committee of the University of Perugia (authorization no. 325/2021-PR; prot. 2B818.83).

**Figure 1:**
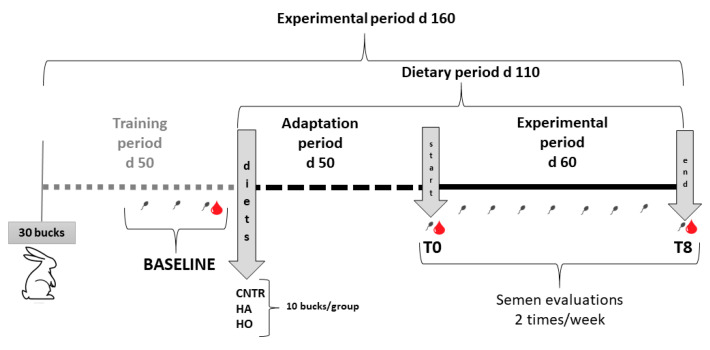
Graphic representation of the experimental plan.

The rabbits were weighed weekly, and the semen was collected twice per week for quality assessments for 12 weeks (3 wks of adaptation + 9 wks for the experimental period; *n* = 24 samples/group). Semen samples collected before the start (baseline) and at the end of the trial (T8, Figure 1), were processed to recover the seminal plasma (SP) and spermatozoa through centrifugation at 2500× *g* for 15 min. Spermatozoa were resuspended in aliquots of 10^8^/mL with phosphate buffer, whereas SP were divided into aliquots of 500 µL and stored at −80 °C for assessment of fatty acid profile and oxidative damage, respectively. For the determination of plasma F_2_-IsoPs, F_3_-IsoPs, and F_4_-NeuroPs, an aliquot of butylhydroxytoluene (BHT) was added (90 μM, final concentration).

Before the start of the experiment (baseline), at T0 and T8, blood samples (2 mL) were obtained from the auricular marginal vein using a 2.5 mL syringe fitted with a butterfly needle, after the local application of an anesthetic cream (EMLA^®^).

Serum was obtained from blood samples coagulated at room temperature for 2 h, and then the collection tubes were rimmed and refrigerated at 4 °C for 24 h until analysis of the FA profile. Plasma was obtained from blood samples collected in tubes containing Na_2_-EDTA, immediately centrifuged at 2500× *g* for 15 min at 4 °C and used for MDA and isoprostane determination. As for the seminal plasma, an aliquot of BHT was added (90 μM, final concentration) for the evaluation F_2-_, F_3_-IsoPs, and F_4_-NeuroPs. 

### 2.2. Semen Quality Assessment

Figure 1 gives the experimental plan.

Semen samples were collected using an artificial vagina heated to 38 °C with water and immediately transferred to the laboratory of Perugia University. The evaluations of sperm quality were immediately performed on raw samples, as reported:

- Volume (mL), which was determined using graduate tubes;
- Sperm concentration (number of sperm × 10^6^/mL), which was measured by means of a Thoma–Zeiss cell counting chamber with a 40× objective;
- Kinetic characteristics, which were analyzed using a Computer-Assisted Semen Analyzer (model ISAS^®^4.0, Valencia, Spain) after appropriate dilution (1/20) with a modified Tyrode’s Albumin Lactate Pyruvate buffer [26] at pH 7.4 and 296 mOsm/kg.

This system consisted of a negative phase-contrast optic system (Olympus CH-2, Steroglass, Bologna, Italy) equipped with a CCD Sony camera. The set-up parameters were previously established, and the acquisition rate was set at 100 Hz [27]. For each sample, two drops and six microscopic fields were analyzed for a total of 300 spermatozoa. Recorded sperm motion parameters were numerous (Table S1), but only the motility rate (percentage of motile sperm/total sperm) and track speed (μm/s, the sum of the incremental distances moved by the sperm in each frame along the sampled path divided by time) were reported.

- Viability of sperm was evaluated under light microscope via the eosin technique according to the WHO [28].

### 2.3. Oxidative Damage of Seminal Plasma and Blood Plasma

Lipid peroxidation in the seminal and blood plasma was assessed by a thiobarbituric reactive substances (TBARs) test by measuring malondialdehyde (MDA) along with other substances that are reactive to 2-thiobarbituric acid (TBA), as reported by Buege et al. [29]. The results were expressed as nmol MDA/mL.

### 2.4. Fatty Acid Profile of Raw Semen and Blood Serum

The lipid extraction from the raw semen and blood serum was performed according to the method of Folch et al. [30], and esterification was carried out following the procedure of Christie [31]. The transmethylation procedure was conducted using heneicosanoic acid methyl esters (Sigma-Aldrich, Steinheim, Germany) as an internal standard. 

The FA composition was determined using a Varian gas-chromatograph (CP-3800) equipped with a flame ionization detector and a capillary column of film (Supelco, Bellefonte, PA, USA). Helium was used as the carrier gas with a flow of 0.6 mL/min. The split ratio was 1:20.

Individual FAME was identified by comparing the relative retention times of peaks in the sample with those of a standard mixture (FAME Mix Supelco; 4:0 to 24:0) plus cis-9 cis-12 C18:2; cis-9 cis-12 cis-15 C18:3; and cis-9 cis-12 cis-15 C18:3 (all from Sigma-Aldrich). The FAs were expressed as % of total FA. The average amount of each FA was used to calculate the sum of the total SFA, monounsaturated fatty acid (MUFA), n-3, and n-6 PUFA. The LC-PUFA included EPA, n-3 DPA, and DHA acids for n-3 and ARA and n-6 DPA acid for n-6.

### 2.5. Free F_2_-IsoPs, F_3_-IsoPs, and F_4_-NeuroPs Determination in Seminal and Blood Plasma

The levels of free F_2_-IsoPs, F_3_-IsoPs, and F_4_-NeuroPs were determined by gas chromatography/negative-ion chemical ionization tandem mass spectrometry (GC/NICI-MS/MS; Thermo Finnigan, San Jose, CA, USA). 

After thawing, the seminal and blood plasma samples were treated with a volume of acidified water (pH 3) and spiked with a tetradeuterated derivative of Prostaglandin F_2_α (PGF_2_α-d4; 500 pg), as an internal standard. Subsequently, solid phase extraction procedures were carried out, according to a previously reported methodology [32]. Briefly, each sample (plasma or seminal plasma) was applied to an octadecylsilane (C18) cartridge, and the eluate was transferred to an aminopropyl (NH_2_) cartridge to collect the isoprostanoids. The final eluates were derivatized to convert the carboxyl group of isoprostanes into pentafluorobenzyl esters and the hydroxyl group into trimethylsilyl ethers, as previously reported [33]. The derivatized isoprostanoids were analyzed by GC/NICI-MS/MS. The ions that were determined were the product ions at *m*/*z* 299 and *m*/*z* 303, derived from the [M-181]—precursor ions of 8-iso-PGF_2_α, also referred to as 15-F_2_t-IsoP (*m*/*z* 569) and PGF_2_α-d4 (*m*/*z* 573), respectively [33], whereas for F_3_-IsoP quantification, the measured ion was the product ion at *m*/*z* 297 derived from the [M-181]− precursor ions (*m*/*z* 567) produced from oxidized EPA.

Concerning F_4_-NeuroPs, the mass ions that were determined were the product ions at *m*/*z* 323 and *m*/*z* 303, derived from the [M-181]-precursor ions of 10-F_4_tNeuroPs, considered as the most represented F_4_-NeuroPs (*m*/*z* 593) [34] and PGF_2_α-d4 (*m*/*z* 573), respectively.

### 2.6. Determination of Cytokines in Seminal Plasma

Some cytokines (IL-6, IL-1b, and TNFα) were detected and quantified in seminal plasma using the Bio-Plex Cytokine assay (Bio-Rad Laboratories S.r.l., Segrate, Milano, Italy) following the manufacturer’s protocols. Briefly, 96-well plates were prewetted with 200 µL of assay buffer (provided by the manufacturer) for 10 min and then aspirated using a vacuum manifold. Standards and seminal plasma (25 µL) were added to appropriate wells, followed by the addition of assay beads. The plates were incubated at RT for 30 min with mild agitation; the fluid was then removed by vacuum, and the wells were washed twice with wash buffer. Detection antibodies were added to each well and incubated for 1 h at RT. The fluorescent conjugate streptavidin–phycoerythrin was added to each well, and the plates were incubated for 30 min at RT. The fluid was then removed by vacuum, and the wells were washed twice. Analysis of each sample was performed in duplicate. The limit of sensitivity was 1.95 pg/mL, and the linear range of detection was 1.95 to 32,000 pg/mL for all the cytokines analyzed in this study. Data were collected and analyzed using a Bio-Plex 200 instrument equipped with BioManager analysis software (Bio-Rad, Bio-Plex manager v5.0, Segrate, Milano, Italy).

### 2.7. Testosterone Evaluation in Blood Plasma

The testosterone concentration in the rabbit serum was performed by radioimmunoassay assay (RIA) using a Testosterone (^125^I) RIA KIT (Ref: RK-61M, Institute of Isotopes Co., Ltd., Bucharest, Hungary). The assay was based on the competition between unlabeled testosterone and a fixed quantity of ^125^I-labelled testosterone for a limited number of binding sites on a testosterone-specific antibody. This allows the reaction of a fixed amount of tracer and antibody with different amounts of unlabeled ligand, with the amount of tracer bound by the antibody being inversely proportional to the concentration of unlabeled ligand. Upon the addition of a magnetizable immunosorbent, the antigen–antibody complex is bound on solid particles, which are then separated by either magnetic sedimentation or centrifugation. Counting the radioactivity of the solid phase enables the construction of a standard curve and samples to be quantitated. The method showed the 100% testosterone, 35% 5α-dihydrotestosterone, 0.8 5β-dihydrotestosterone, 0.01 17β-estradiol, and 0.01 cortisol cross-reactions. The sensitivity was the lowest dose of testosterone that was 5% lower than the initial binding capacity.

### 2.8. Statistical Analysis 

The lipid oxidation, fatty acid profile, and sperm kinetic traits were analyzed using a linear model to evaluate the fixed effect of diet, time, and their interaction (CNT, HA, HO; SPSS v28). Free isoprostanoids, testosterone, and cytokines were analyzed using a linear model with diet as a fixed effect. 

The least squares (LS) mean, pooled standard error (SE), and Root Mean Square Error (RMSE) are reported. The Bonferroni correction was applied for multiple comparisons. The significance was set at *p* ≤ 0.05. Regression curves were estimated with linear and non-linear models using R^2^ as an estimate of the goodness-of-fit.

## 3. Results

### 3.1. Experimental Diets

Table 2 reports the fatty acid profile of the rabbit diets. The addition of coconut oil to the standard diet increased the content of SFA (23.42 vs. 14.50 and 10.45% of total FA in HA and CNT, HO, respectively), mainly due to the higher content of lauric (C12:0), palmitic (C16:0), and myristic (C14:0) acids. Conversely, the linseed oil provided a higher amount of ALA (18.63% vs. 11.15% and 1.08% in HO, CNT, and HA, respectively). 

The PUFA content was higher in the CNT and HO diets compared to HA (56.51%, 53.26% vs. 39.54%, respectively).

### 3.2. Semen Quality Parameters

**Figure 2:**
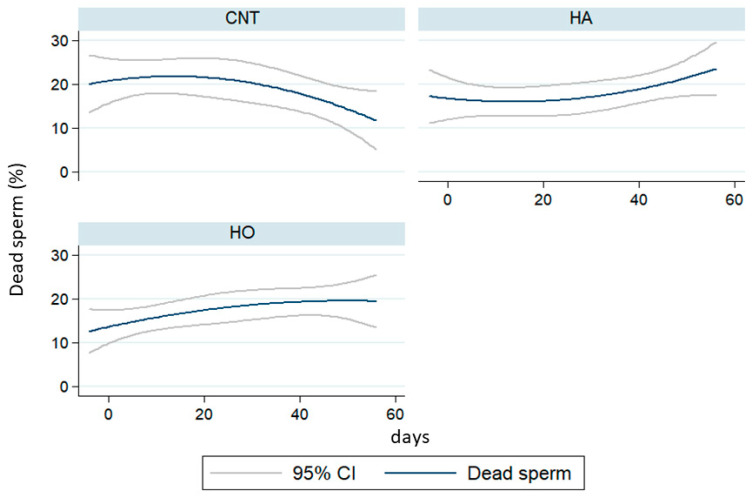
Dead sperm (%) of rabbits fed the control (CNT), high-atherogenic (HA), and high-oxidative (HO) diets (95% upper and lower confidence intervals).

**Figure 3:**
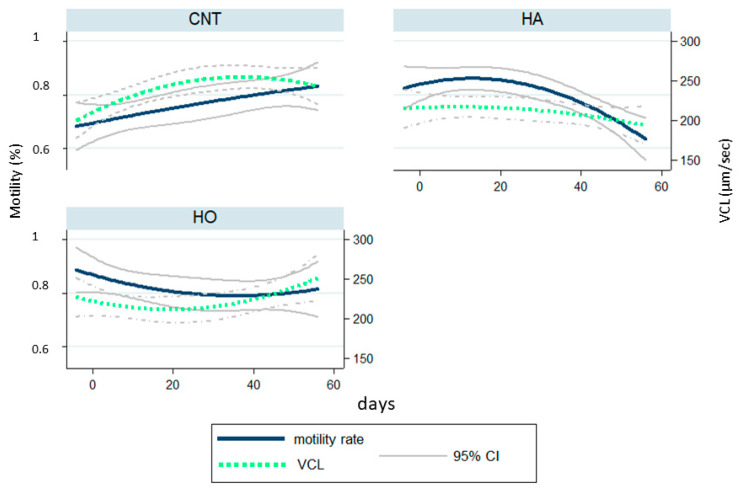
Sperm curvilinear velocity (VCL, µm/s) and motility rate (%) of rabbits fed the control (CNT), high-atherogenic (HA), and high-oxidative (HO) diets (95% upper and lower confidence intervals).

The quality parameters of semen are reported in Table S1, Figure 2 and Figure 3. The main differences were observed after 50 days of dietary treatments, with a reduction of sperm viability in both the HO and HA groups (Figure 2). Similarly, the proportion of motile cells (Figure 3) was reduced after 60 days of feeding the experimental diets (T8), mainly in the HA group (18% vs. 44% and 28% in CNT, HA, and HO, respectively; Table S1), whereas the HO group showed a decline after 10–14 days (T1–T2, Table S1).

The kinetic parameters (Figure 3) showed a worsening trend in the HA, starting from 30 days (T5, Table S1), with a significantly lower value in curvilinear velocity (VCL) compared to the CNT and HO groups after 60 days of diet administration. 

### 3.3. Oxidative Damage and Fatty Acid Profile of Semen 

#### 3.3.1. Oxidative Damage of Semen

Lipid oxidation of seminal plasma is reported in Table 3. The different dietary groups showed a significant difference in MDA content after 50 days of dietary administration (baseline vs. T0, *p* = 0.050). Both HA and especially HO showed higher values of MDA at T8 (31.31 vs. 24.82 vs. 17.14 nmol MDA/mL, in HO, HA, and CNT, respectively).

#### 3.3.2. Fatty Acid Profile of Rabbit Semen

Table 3 reports the fatty acid profile of raw semen. The data showed that the HO group had a higher ALA content compared to the other groups at every time point; however, this concentration seemed to decrease over time. 

Significance was also found for C20:2n-6 and total n-6 PUFA where the highest proportion was recorded in CNT. DHA and n-3 PUFA were also higher (*p* > 0.05) in the HO group compared to the other diets, but the high variability of the analytical determinations rendered the differences non-significant. 

### 3.4. Blood Analytical Determination

#### 3.4.1. Oxidative Damage of Blood Plasma

Lipid oxidation of blood plasma is reported in Table 4. No significant differences were found among dietary groups.

#### 3.4.2. Fatty Acid Profile of Blood Serum

Table 4 reports the fatty acid profile of blood serum. A higher proportion (*p* ≤ 0.05) of myristic (C14:0) and margaric (C17:0) acid was found in the HA group at T8. Furthermore, a higher value of DHA in the serum of the rabbits fed flaxseed oil was found at T8.

#### 3.4.3. Isoprostanoids in Seminal and Blood Plasma 

In Table 5, the isoprostanoids content of seminal and blood plasma of rabbits at the start and end of the experimental period are given. In seminal plasma, the isoprostanoids of rabbits fed pro-oxidant (HO) and pro-inflammatory (HA) diets did not show significant differences compared to the baseline. However, F_2_-IsoPs were higher in the CNT group. Similarly, the F_2_-IsoPs of blood plasma were higher in CNT with respect to the other dietary groups and baseline; conversely, the F_3_-IsoPs and F_4_-NeuroPs of the HO and HA groups showed higher values than CNT.

#### 3.4.4. Blood Testosterone and Cytokines in Seminal Plasma

The testosterone content (Table 6) of blood was significantly higher in the HA groups, followed by HO and CNT. Table 6 also reports the cytokine concentration of the blood. The only difference was recorded in the TNF-α levels, where HA showed the highest value (0.10 vs. 0.05 pg/mL HA vs. both HO and CNT). 

## 4. Discussion

The results obtained from the present investigation suggested that there is a close relationship between the nature of the fatty acids administered through the diet and the triggering of an oxidative and/or inflammatory response in the blood and semen of rabbit. 

Oxidative stress occurs when the generation of free radicals and other active intermediates of biological systems exceed the antioxidants’ ability to neutralize and eliminate them [35,36]. The current concept of “oxidative stress” includes the pathways related to “nitrosative stress” (Reactive Nitrose Substances—RNS production) and “metabolic stress” (Reactive Oxygen substances—ROS production). Under these conditions, ROS and RNS act as “toxic” substances that may react with proteins, carbohydrates, and lipids resulting in alterations in intra- and extra-cellular homeostasis, leading to possible cell death and regeneration [37,38].

**Figure 4:**
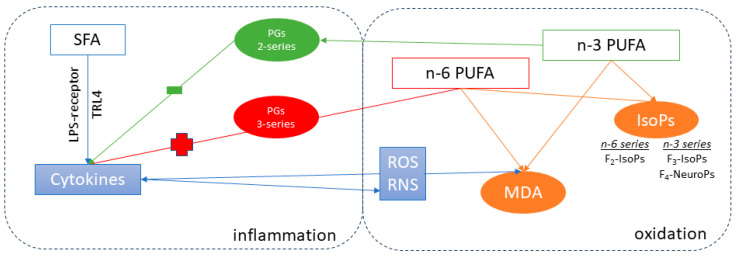
Simplified pattern of the inflammation–oxidation relationship. SFA induces the release of some cytokines (i.e., TNF-α) by increasing endotoxin release (LPS), starting the process of cell inflammation. Both PUFA series induce oxidation, along with MDA and isoprostanoid production. Furthermore, PUFA mediators play a crucial role in the regulation of inflammation: 3-series PGs, derived from n-6 PUFA, enhance tissue inflammation (red color), whereas 2-series PGs (n-3 PUFA) reduce inflammation (green color). The resulting increase in ROS and RNS, due to cytokine modulation, induces an additional inflammatory and oxidative response.

However, the oxidative process is strictly connected to the inflammatory status (see Figure 4). Indeed, inflammatory cells produce a large number of cytokines and chemokines that promote ROS and RNS release through the activation of protein–kinases signaling [35,39]. For example, TNF-α enhances the formation of ROS by neutrophils and other cell lines, while interleukin-1β (IL-1β) and interferon (IFN)-γ stimulate the expression of inducible nitric oxide synthase in inflammatory cell lines [40]. Furthermore, the production of IL-6 may be stimulated by PGs (mainly of 3-series) derived from n-6 PUFA (ARA), which is elevated in inflammatory macrophages [41,42]. 

In this complex mechanism, the lipid and sugar contents of the diet [10] and the fatty acid profile of the tissue play a crucial role. Indeed, unhealthy hypercaloric diets or foods with a high glycemic index may be directly associated with increased oxidative stress as occurs in many degenerative diseases: e.g., cancer, especially colon and breast, cardiovascular and metabolic diseases, including metabolic syndrome, type II diabetes, and in the liver, non-alcoholic fatty liver disease (NAFLD) [43]. It is well known that PUFA can stimulate oxidation due to the unsaturation in the hydrocarbon chain: double bonds can be attached to by ROS forming aldehydes [44,45]. Furthermore, PUFA are also exposed to free-radical-induced peroxidation due to the esterification of FAs in cell membrane phospholipids, in turn generating isoprostanoids [18]. 

In the present research, the main driver of the oxidative mechanisms of tissues seemed to be the PUFAs profile, because the dietary energy supply of the diets was similar (12.06, 11.86, and 11.88 MJ/kg in CNT, HA, and HO, respectively, Table 1). The literature has reported that SFA directly affects cell inflammation by promoting the translocation of microbiome products (>100 trillion) from the gut into the bloodstream. Between them, lipopolysaccharide (LPS) is considered an endotoxin, because it is an endogenous component of the cell wall of all Gram-negative bacteria [46]. Apart from the role of SFAs in promoting the uptake of endotoxins, many researchers believe that dietary SFAs directly affect inflammation. For example, it has long been known that SFAs are essential structural components of bacterial endotoxins (lipid A [47]). Accordingly, the substitution of SFAs with MUFAs or PUFAs reduces the proinflammatory activity of LPS. Macrophages, and other cells of the innate immune system, possess receptors (i.e., toll-like receptor, TLR4) that recognize LPS [47,48]. LPS-mediated signaling through TLR4 leads to the activation of NF-kB, a transcription factor, that subsequently turns on the expression of numerous proinflammatory cytokines, such as TNF-α, IL-1, IL-6, and IL-8 [41].

In the present research, the trend of oxidation markers and cytokines in the HA and HO diets underlined the strict connection between inflammation and oxidation. Accordingly, the expected improvement of semen traits due to n-3 PUFA supplementation was lacking, probably as the rabbits of the HO group were more vulnerable (right side of Figure 4) to oxidation due to the higher unsaturation of this diet. It should be noted that there was no additional antioxidant protection in any of the diets [49,50]; many researchers have confirmed that the addition of vitamin E to n-3 enriched diets is a prerequisite to prevent the susceptibility of spermatozoa to lipid peroxidation [24]. Indeed, sperm cells contain about 50% PUFA [16] in their membrane and a low concentration of cytosolic scavenger enzymes [51] thus requiring additional protection. Our previous results showed that the oral administration of n-3 PUFA, furnished by flaxseed or fish oil, and protected by α-tocopheryl acetate addition (200 mg/kg of diet) exerted a positive effect of the reproductive activity of rabbit bucks [24,52], indirectly confirming our hypothesis. 

The administration of pro-oxidant (HO) and pro-atherogenic (HA) diets basically increased (*p* = 0.050) the lipid oxidation (MDA) of rabbit seminal plasma whereas, F_2_-IsoPs (derived from ARA) increased only in the CNT group. Conversely, the MDA did not change in the blood plasma, but a significant increase of F_4_-NeuroPs (derived from DHA) and F_3_-IsoPs (derived from EPA) was recorded in HO and especially in the HA group. Similarly, Logini et al. [53] found no differences in the F_4_-NeuroPs value in the semen of men of varicocele group (presence of inflammatory status in testis) with respect to healthy fertile men.

Further studies are needed to understand the correlation between SFA and F_4_-NeuroPs; probably, the differences recorded in the blood are ascribable to the fact that blood is less protected than semen, considering that the testis has the Blood Testis Barrier (BTB) to protect sperm from inflammation/oxidation/damage [54].

The FA profile of the blood showed higher concentration of ARA in CNT than in HO and HA, justifying the increase in F_2_-IsoPs. Similarly, DHA was higher in HO but not in HA, partially explaining the trend of F_4_-NeuroPs. It is likely that the higher susceptibility of DHA to oxidation (six double bonds on the hydrocarbon chain) makes it a preferential substrate in high-inflammatory conditions, such as those sustained by the HA group, similar to the oxidation of low-density lipoprotein (LDL) [55], which mainly occurs when diets are enriched in SFA. 

In our previous papers, we demonstrated the strict relationship between isoprostanoids in the semen [17], brain, and testes of rabbits and the FA profile (ARA, EPA, or DHA): diets rich in n-3 PUFA increased F_3_-IsoPs [24] and F_4_-NeuroPs. The body of literature reported that isoprostanoids are markers for several neurodegenerative (Parkinson’s and Alzheimer’s disease) [56,57] disorders; in particular, F_4_-NeuroPs are considered markers of structural and functional injuries of cells and apparatus. Indeed, the decline in integrity of this tissue seems correlated with the loss of DHA in membranes and the increase in F_4_-NeuroPs [57]. However, as affirmed above, their abundance also may be modulated by dietary intake. 

In agreement with the literature, the present findings confirmed that coconut oil (HA) provides a higher quantity of SFA; however, no differences in the SFA profile of the sperm or blood were recorded. It is probable that HA administration, even if not enough to change the FA profile, was pro-inflammatory, activating LPS uptake. Mani et al. [58] demonstrated that pigs fed coconut oil had the highest concentration of blood endotoxins followed by those fed vegetable and fish oil. 

In 2001, Lee et al. [59] were the first to demonstrate that SFAs were able to directly stimulate inflammatory gene expression by TLR4 signaling. The relative proinflammatory effectiveness of various SFAs reduces as the chain length increases: lauric acid (12:0) > myristic acid (14:0) > stearic acid (18:0). In the present research, we found an increase of TNF-α level only in the HA group, confirming the pro-inflammatory effects of coconut oil.

However, many contrasting results have been reported in the literature, possibly related to the species analyzed and to the experimental design. Himashu et al. [60] found that rams fed coconut (5%) or linseed (5%) oils showed higher antioxidant enzyme activity in the semen and lower oxidative damage with respect to the control, with no differences in the kinetic traits of semen.

Our results demonstrated that the administration of HA and HO diets increased the percentage of dead sperm with a similar trend over time, whereas the kinetic parameters (VCL) largely changed. In particular, HA showed a drastic reduction of motility traits after 50 days of feeding (over 110 days of dietary treatments). Conversely, HO bucks showed a strong fall of VCL after 10 days of experimentation (60 days of dietary treatments) with ongoing recovery and a low reduction of motility rate. 

Under physiological conditions, ROS regulate the sperm mechanisms involved in capacitation, hyperactivation, acrosome reaction, and fertilization. However, if the ROS level is over the physiological threshold, a condition of oxidative stress leads to lipid peroxidation, DNA damage, and protein oxidative damage [61]. Many papers have reported a negative correlation between ROS (oxidation) and the kinetic parameters of sperm. 

The data herein reported suggest that the HA diet interfered with spermatogenesis, causing structural damage induced by an excess of ROS release, as it influenced the sperm motility of the new sperm wave (VCL decrease over time). Conversely, HO showed an acute effect, probably due to a membrane change: the different FA profile modulated the capacitation of sperm, and consequently VCL increased [62,63]. Accordingly, it is possible to assume that the oxidative thrust (i.e., cytokines release) disturbed the spermatozoa environment (i.e., seminal plasma), but did not cause any damage to Leydig or Sertoli cell [64].

Furthermore, in agreement with other authors [60,65], the HA group showed an increase in testosterone concentration, followed by HO, probably due to the higher steroid synthesis influenced by SFAs. Several papers have reported a correlation between dietary coconut administration and spermatogenesis, mainly due to the enhancement of cholesterol metabolism; such a change is likely to be partly modulated by fat content. Indeed, the intake of high concentrations of lipids stimulates the GnRH secretion along with an increase of Leydig cells in the testes [66,67]. 

## 5. Conclusions

Pro-atherogenic (HA-coconut oil) and pro-oxidative (HO-linseed oil) diets affect the inflammatory and oxidative response of rabbit bucks, with the main effect occurring in the blood plasma. The resulting outcomes were mainly attributable to the different fatty acid profiles (SFA vs. PUFA), which triggered an inflammatory/oxidative response. Indeed, the dietary fatty acid profile induced modifications in the motility and viability of sperm and the oxidation of semen, whereas the fatty acid profile was not affected.

Furthermore, the present results confirmed the importance of antioxidant protection when a PUFA-enriched diet is administered (i.e., linseed), in order to minimize the negative effects of oxidation and promote the benefits of n-3 PUFA. In this regard, it is important to understand if/which adequate intakes of antioxidant molecules are able to counteract reproductive problems, or whether it is necessary to intervene with anti-inflammatory molecules to address a full-blown pathological state.

Further studies are needed to understand the impact of unprotected lipids on the testes and other relevant tissues (the brain, as a tissue rich in n-3 PUFA, and the liver, as the lipid metabolic center), better deepening the role of some specific pro-oxidant (e.g., hydroperoxides, aldehydes, etc.) and antioxidant (vitamin E, C, enzyme antioxidants) molecules. 

## Figures and Tables

**Table 1:** Formulation and proximate analysis of the control (CNT), high-atherogenic (HA), and high-oxidative (HO) diets.

Ingredients	Units	CNT	HA	HO
Wheat bran	g/kg	410	300	300
Sunflower meal	g/kg	170	170	170
Dehydrated alfalfa meal 17%	g/kg	280	188	188
Barley meal	g/kg	58	70	70
Dry beet pulp	g/kg	-	70	70
Wheat straw	g/kg	-	50	50
Beet molasses	g/kg	20	30	30
Soybean meal 44%	g/kg	30	60	60
Flaxseed oil *	g/kg	-	-	30
Coconut oil **	g/kg	-	30	-
Calcium carbonate	g/kg	16	16	16
Vitamin–mineral premix †	g/kg	10	10	10
Salt	g/kg	3.8	3.8	3.8
Dl-methionine	g/kg	2.2	2.2	2.2
**Proximate composition, d.m. basis**				
Crude protein	g/kg	18.95	18.75	18.88
Ether extract	g/kg	4.10	5.63	5.64
NDF	g/kg	35.26	35.63	35.63
ADF	g/kg	20.25	20.36	20.83
ADL	g/kg	5.33	4.93	5.04
Ash	g/kg	10.01	9.31	9.00
Estimated digestible energy ^§^	MJ/kg	12.06	11.86	11.88

* 100% of flaxseed oil (Bleu&Marine Bretania Cosmetics, Alhaurín de la Torre, France) containing 60% of α-linolenic acid. ** coconut oil (CocoNativo Bio, Monte Nativo, Italy): 95% of saturated fatty acids. † Per kg diet: vitamin A—11,000 IU; vitamin D3—2000 IU; vitamin B1—2.5 mg; vitamin B2—4 mg; vitamin B6—1.25 mg; vitamin B12—0.01 mg; alpha-tocopheryl acetate—200 mg; biotin—0.06 mg; vitamin K—2.5 mg; niacin—15 mg; folic acid—0.30 mg; D-pantothenic acid—10 mg; choline—600 mg; Mn—60 mg; Fe—50 mg; Zn—15 mg; I—0.5 mg; Co—0.5 mg. ^§^ Estimated by Maertens et al. [25].

**Table 2:** Fatty acids profile (% of total FA, mean ± SE) of the control (CNT), high-atherogenic (HA), and high-oxidative (HO) diets.

Fatty Acids	CNT	HA	HO
C12	tr *	8.84 ± 2.36	0.06 ± 0.03
C14	0.25 ± 0.01	3.74 ± 1.05	0.11 ± 0.04
C15	tr	0.02 ± 0.01	tr *
C16	8.95 ± 1.12	7.58 ± 0.22	6.77 ± 0.47
C16:1	0.25 ± 0.02	0.10 ± 0.06	0.18 ± 0.04
C17	0.05 ± 0.03	0.04 ± 0.01	0.06 ± 0.01
C17:1	0.05 ± 0.03	0.02 ± 0.01	0.04 ± 0.01
C18	5.25 ± 1.01	3.20 ± 0.03	3.44 ± 0.22
C18:1n-9	23.25 ± 3.25	33.53 ± 8.23	31.99 ± 6.43
C18:2n-6, LA	45.36 ± 6.12	38.45 ± 6.36	34.63 ± 7.14
C18:3n-3, ALA	11.15 ± 2.14	1.08 ± 0.65	18.63 ± 2.04
others	5.44 ± 0.96	3.38 ± 0.14	4.08 ± 1.22
SFA	14.50 ± 3.45	23.42 ± 3.47	10.45 ± 0.77
MUFA	23.55 ± 6.07	33.66 ± 6.41	32.21 ± 11.45
PUFA	56.51 ± 11.47	39.54 ± 10.08	53.26 ± 16.32

* tr: trace.

**Table 3:** Lipid oxidation (nmol MDA/mL of seminal plasma) and semen fatty acid profile (% of total FA) of rabbits fed the control (CNT), high-atherogenic (HA), and high-oxidative (HO) diets.

		T0			T8				Significance		
	BASELINE	CNT	HA	HO	CNT	HA	HO	RMSE	Group (G)	Time (T)	G × T
MDA (nmol/mL)	16.76	20.36	23.44	22.03	17.14	24.82	31.31	1.20	0.050	0.066	0.305
C12	0.13	0.16	0.00	0.10	0.13	0.42	0.18	0.17	0.858	0.034	0.052
C14	9.05	8.16	9.95	6.39	8.75	10.00	9.21	0.74	0.319	0.791	0.950
C16	15.71	15.74	14.57	18.79	15.63	16.73	15.32	0.61	0.470	0.858	0.284
C16:1	0.23	0.20	0.18	1.33	0.26	0.12	0.10	0.38	0.199	0.549	0.649
C18	19.71	19.89	17.51	15.98	19.71	18.35	18.87	0.70	0.049	0.498	0.476
C18:1n-9	11.00	11.33	10.55	20.33	10.95	11.03	8.44	1.29	0.387	0.499	0.551
C18:2n-6, LA	7.86	7.46	10.91	12.08	8.51	9.67	11.17	0.80	0.301	0.883	0.977
C18:3n-3, α-ALA	0.35 ^b^	0.34 ^b^	0.22 ^ab^	0.96 ^c^	0.39 ^b^	0.16 ^a^	0.46 ^b^	0.33	0.782	0.285	0.002
C20:2n-6	2.63 ^c^	2.06 ^b^	1.53 ^b^	0.16 ^a^	2.00 ^b^	0.25 ^a^	0.41 ^a^	0.46	0.000	0.095	0.028
C20:3n-6	0.85	0.86	0.66	0.73	0.41	0.74	0.90	0.22	0.810	0.599	0.333
C20:4n-6, AA	1.39	1.55	1.26	1.01	1.35	1.19	1.22	0.32	0.427	0.291	0.056
C20:5n-3, EPA	2.81	2.41	1.94	1.94	1.53	1.54	2.13	0.34	0.107	0.243	0.108
C22:5n-6, DPAn-6	19.04	19.19	14.74	12.48	18.89	15.68	17.27	1.02	0.339	0.796	0.786
C22:6n-3, DHA	0.53	0.25	0.00	0.85	0.06	0.16	0.22	0.30	0.610	0.676	0.491
Others	6.73	8.03	15.99	10.87	11.43	14.06	17.09	1.00	0.690	0.807	0.328
SFA	44.59	43.94	42.02	41.25	44.22	45.51	43.59	0.88	0.322	0.655	0.742
MUFA	11.23	11.53	10.73	21.66	11.21	11.15	8.54	1.15	0.350	0.508	0.563
PUFA	37.45	34.80	31.26	29.72	33.15	30.68	34.64	0.83	0.139	0.809	0.132
n-3	4.53	3.85	2.82	4.49	2.40	2.60	3.71	0.42	0.791	0.433	0.149
n-6	33.76 ^b^	33.11 ^b^	29.10 ^b^	22.47 ^a^	31.16 ^b^	27.43 ^a^	26.97 ^a^	0.80	0.048	0.595	0.050

^a–c^ on the same row means *p* ≤ 0.05 for G × T. RMSE: Root Mean Square Error.

**Table 4:** Lipid oxidation (nmol MDA/mL of blood plasma) and plasma fatty acid profile (% of total FA) of rabbits fed the control (CNT), high-atherogenic (HA), and high-oxidative (HO) diets.

		T0			T8				Significance		
	BASELINE	CNT	HA	HO	CNT	HA	HO	RMSE	Group (G)	Time (T)	G × T
MDA (nmol/mL)	8.24	8.26	11.35	9.68	9.79	10.96	11.25	0.62	0.147	0.486	0.916
C12	0.03	0.02	0.84	0.00	0.06	0.55	0.00	0.28	0.000	0.208	0.338
C14	0.70 ^b^	0.65 ^b^	1.30 ^c^	0.25 ^a^	0.59 ^b^	1.10 ^c^	0.42 ^a^	0.28	0.000	0.001	0.002
C15	0.29	0.48	0.36	0.18	0.24	0.28	0.23	0.16	0.274	0.442	0.443
C16	22.91	21.24	15.72	13.82	19.76	14.48	17.63	1.10	0.029	0.210	0.376
C16:	1.35	1.04	0.80	0.34	1.11	0.72	1.12	0.36	0.291	0.456	0.720
C17	0.66 ^c^	0.68 ^c^	0.51 ^b^	0.57 ^bc^	0.39 ^a^	0.53 ^b^	0.44 ^ab^	0.18	0.300	0.006	0.033
C18	12.74	13.14	13.00	13.40	13.10	14.18	11.66	0.47	0.307	0.705	0.276
C18:1n-9	17.65	20.18	21.90	21.34	18.63	20.11	25.64	0.90	0.388	0.915	0.686
C18:2n-6, LA	25.76	26.84	34.32	36.19	24.21	36.58	27.17	1.13	0.023	0.110	0.331
C18:3n-3, α-ALA	0.92	0.32	0.50	3.75	0.39	0.46	2.48	0.52	0.000	0.311	0.229
C20:4n-6, AA	2.64	2.25	1.99	1.93	2.04	1.87	1.69	0.29	0.044	0.279	0.690
C20:5n-3, EPA	0.21	0.12	0.15	0.09	0.13	0.13	0.20	0.11	0.809	0.277	0.430
C22:5n-6, DPAn-6	0.26	0.25	0.15	0.11	0.16	0.17	0.09	0.12	0.000	0.058	0.075
C22:5n-3, DPAn-3	0.08	0.21	0.12	0.05	0.13	0.04	0.05	0.13	0.006	0.115	0.777
C22:6n-3, DHA	0.24 ^b^	0.22 ^b^	0.17 ^ab^	0.33 ^b^	0.13 ^a^	0.18 ^ab^	0.66 ^c^	0.43	0.001	0.007	0.002
SFA	37.33	36.22	31.73	28.22	34.14	31.12	30.37	1.08	0.028	0.194	0.613
MUFA	19.05	21.25	22.76	21.72	19.80	20.87	26.79	0.93	0.578	0.858	0.676
PUFA	30.22	30.28	37.65	42.71	27.37	39.70	32.48	1.19	0.037	0.120	0.420
n-3	1.45	0.87	0.94	4.20	0.77	0.80	3.39	0.60	0.000	0.672	0.900
n-6	28.77	29.41	36.71	38.51	26.59	38.90	29.09	1.13	0.033	0.099	0.321

^a–c^ on the same row means *p* ≤ 0.05 for G × T. RMSE: Root Mean Square Error.

**Table 5:** Isoprostanes (pg/mL) in the seminal and blood plasma of rabbits fed the control (CNT), high-atherogenic (HA), and high-oxidative (HO) diets.

	BASELINE	CNT	HA	HO	RMSE	*p* Value
Seminal plasma						
F_2_-IsoPs	87.90 ^a^	124.05 ^b^	95.55 ^a^	75.05 ^a^	0.13	0.009
F_3_-IsoPs	7.45	7.53	8.83	9.16	0.59	0.110
F_4_-NeuroPs	15.67	14.55	22.18	18.90	0.35	0.246
Blood plasma						
F_2_-IsoPs	91.54 ^a^	150.20 ^b^	113.35 ^a^	106.71 ^a^	0.64	0.003
F_3_-IsoPs	5.99 ^a^	6.12 ^a^	8.96 ^b^	8.45 ^b^	0.78	0.034
F_4_-NeuroPs	10.35 ^a^	8.55 ^a^	17.47 ^b^	15.24 ^b^	0.67	0.039

^a–b^ on the same row means *p* < 0.05. RMSE: Root Mean Square Error.

**Table 6:** Blood serum testosterone and seminal plasma cytokines (pg/mL) of rabbits fed the control (CNT), high-atherogenic (HA), and high-oxidative (HO) diets.

	BASELINE	CNT	HA	HO	RMSE	*p* Value
Testosterone	3.02 ^a^	3.12 ^a^	5.62 ^c^	4.89 ^b^	0.205	0.042
IL-1β	29.04	28.47	25.51	29.21	1.125	0.146
TNF-α	0.04 ^a^	0.05 ^a^	0.10 ^b^	0.05 ^a^	0.01	0.005
IL-6	0.35	0.38	0.31	0.33	0.03	0.584

^a–c^ on the same row means *p* ≤ 0.05. RMSE Root Mean Square Error.

## Data Availability

The data presented in this study are available on request from the corresponding author. The data are not publicly available due to the agreement of the project associated.

## Supplementary Materials

The following supporting information can be downloaded at: https://www.mdpi.com/article/10.3390/antiox12101880/s1, Table S1: Semen quality of rabbits fed control (CNT), high-atherogenic (HA), and high-oxidative (HO) diets.
