# Supplementary material for: Pro-Atherogenic and Pro-Oxidant Diets Influence Semen and Blood Traits of Rabbit Bucks
Source: Antioxidants (Basel). 2023 Oct 19;12(10):1880. doi: 10.3390/antiox12101880 (PMC10603835; doi:10.3390/antiox12101880)
Supplement: Supplementary file 1 [file antioxidants-12-01880-s001.zip › antioxidants-2611217-supplementary.pdf]

Table S1. Semen quality of rabbit fed control (CNT), High Atherogenic (HA) and High Oxidative (HO) diets.

		Volume, mL	concentration, n. x 10E06	Dead %	Static %	No mobile progressive %	Mobile progressive %	VCL, µm/s	VSL, µm/s	VAP, µm/s	LIN, %	ALH, µm	BCF, Hz
	CNT	0.33	395.31	46.55	0.3	0.45	0.25	159.86	37.5	64.69	24.38	3.62	12.44
	HA	0.13	413.67	35.83	0.15	0.58	0.26	158.89	39.17	69.12	25.73	3.51	12.6
baseline	HO	0.46	456.33	17.33	0.09	0.55	0.37	185.3	48.19	76.79	25.32	4.45	14.24
	CNT	0.20	550.21	40.00	0.48	0.39	0.14	164.34	32.34	62.24	19.61	3.24	11.67
	HA	0.49	493.27	9.00	0.06	0.68	0.25	270.17	64.65	138.89	24.03	3.51	29
T0	HO	0.44	492.87	12.00	0.19	0.74	0.07	212.93	39.59	102.92	18.27	3.02	17.56
	CNT	0.15	502.41	32.60	0.38	0.49	0.14	206.02	45.48	92.76	21.95	3.47	14.85
	HA	0.33	711.42	26.88	0.31	0.55	0.14	171.12	36.69	82.45	21.74	2.03	14.43
T1	HO	0.53	667.88	14.38	0.08	0.78	0.13	219.97	44.16	108.13	20.36	2.70	21.11
	CNT	0.31	661.00	29.50	0.15	0.70	0.15	262.3	57.46	131.17	21.81	3.11	19.80
	HA	0.51	605.84	12.40	0.12	0.73	0.14	238.37	48.63	113.75	20.66	2.93	23.12
T2	HO	0.34	503.68	15.13	0.11	0.65	0.23	209.43	47.88	102.21	22.95	3.00	24.06
	CNT	0.43	550.64	21.2	0.17	0.69	0.14	256.69	51.75	123.91	20.1	3.43	18.63
	HA	0.55	459.48	15.9	0.16	0.59	0.26	219.18	52.07	103.86	23.75	3.12	26.50
T3	HO	0.45	638.92	20.88	0.28	0.59	0.13	190.73	39.58	93.65	20.82	2.58	21.91
	CNT	0.26	461.38	21.50	0.50	0.42	0.08	211.34	38.26	97.87	18.04	2.62	10.58
	HA	0.28	470.77	23.10	0.19	0.67	0.14	191.90	39.12	88.95	20.33	2.51	18.60
T4	HO	0.50	272.22	20.50	0.35	0.47	0.18	192.67	43.29	84.04	22.53	2.48	15.46
	CNT	0.23	437.90	12.67	0.17	0.69	0.14	242.09	48.15	116.16	19.79	3.32	18.95
	HA	0.42	592.74	11.00	0.16	0.73	0.11	239.76	46.4	118.95	19.51	3.11	18.90
T5	HO	0.66	550.85	17.17	0.19	0.71	0.10	273.54	50.45	136.11	18.43	3.35	22.84

	CNT	0.41	456.93	9.50	0.23	0.67	0.10	250.44	50.32	126.49	19.61	2.94	18.21
	HA	0.35	704.26	17.2	0.21	0.65	0.15	184.26	39.12	85.94	21.28	2.60	19.72
T6	HO	0.69	600.78	18.5	0.14	0.70	0.16	225.54	48.20	118.76	21.27	2.80	23.94
	CNT	0.53	536.59	10	0.15	0.78	0.07	307.33	54.05	162.09	17.81	3.62	14.42
	HA	0.35	470.77	23.00	0.26	0.63	0.12	212.61	46.08	106.29	21.70	2.68	20.21
T7	HO	0.40	709.84	17.5	0.1	0.75	0.15	228.49	52.72	123.08	22.47	2.98	24.09
	CNT	0.53	447.22	18.50	0.18	0.74	0.09	244.85	45.23	118.88	18.52	3.03	17.05
	HA	0.34	670.53	25.00	0.44	0.49	0.07	188.64	35.88	96.74	19.22	2.64	10.81
T8	HO	0.67	634.00	21.50	0.28	0.61	0.11	248.21	43.35	127.85	17.33	3.1	22.97
	RMSE	0.147	4.355	1.044	0.119	0.116	0.097	2.047	1.02	1.558	0.588	0.272	0.794
	Group	0.003	0.410	0.011	0.012	0.157	0.208	0.016	0.851	0.053	0.143	0.110	0.001
	Time	0.330	0.240	<0.001	0.005	0.001	<0.001	<0.001	0.121	<0.001	0.010	0.026	0.004
Sign.	Group x Time	0.196	0.674	<0.001	<0.001	<0.001	0.312	<0.001	0.019	<0.001	0.766	0.763	0.059

VCL: curvilinear velocity; VSL: Linear Velocity; VAP: Average Path Velocity; LIN: linearity; ALH: amplitude of lateral head displacement; BCF: beat cross frequency; RMSE: Root Mean Standard Error
